# A scoping review protocol to identify clinical signs, symptoms and biomarkers indicative of biofilm presence in chronic wounds

**DOI:** 10.12688/hrbopenres.13300.1

**Published:** 2021-07-08

**Authors:** John D. Ivory, Akke Vellinga, James O'Gara, Georgina Gethin

**Affiliations:** 1 School of Nursing & Midwifery, National University of Ireland, Galway, Galway, Galway, H91TK33, Ireland; 2 Alliance for Research & Innovation in Wounds (ARIW), National University of Ireland, Galway, Galway, Galway, H91TK33, Ireland; 3 CDA Diabetic Foot Disease: from PRevention to IMproved patient Outcomes (CDA DFD-PRIMO) Program, National University of Ireland, Galway, Galway, Galway, H91TK33, Ireland; 4 Irish Research Council, 3 Shelbourne Buildings, Crampton Avenue, Ballsbridge, D04 C2Y6, Ireland; 5 School of Medicine, National University of Ireland, Galway, Galway, Galway, H91TK33, Ireland; 6 Microbiology, School of Natural Sciences, National University of Ireland, Galway, Galway, Galway, H91TK33, Ireland; 7 School of Nursing and Midwifery, Monash University, Melbourne, Australia

**Keywords:** Chronic wound, Wound healing, Infection, Biofilm, Clinical signs and symptoms

## Abstract

**Introduction:** Wound healing is characterised by haemostatic, inflammatory, proliferative and remodelling phases. In the presence of comorbidities such as diabetes, healing can stall and chronic wounds may result. Infection is detrimental to these wounds and associated with poor outcomes. Wounds are contaminated with microbes and debris, and factors such as host resistance, bacterial virulence, species synergy and bioburden determine whether a wound will deteriorate to critically colonised/infected states. Biofilms are sessile microbial communities, exhibiting high-level antibiotic tolerance and resistance to host defences. Biofilm in critically colonised wounds can contribute to delayed healing. Little is known about clinical presentation and diagnosis of wound biofilms.

**Objective:** To identify from the literature clinical signs, symptoms and biomarkers that may indicate biofilm in chronic wounds.

**Methods:** This review will be guided by the Preferred Reporting Items for Systematic Reviews extension for Scoping Reviews (PRISMA-ScR), and the Joanna Briggs Institute Manual for Evidence Synthesis. Studies of any design in any language recruiting adult patients with  venous, diabetic, pressure or mixed arterial-venous ulcers and reporting data on clinical signs/symptoms of biofilm are eligible. Searches of Medline, Embase, CINAHL, Cochrane Central, Scopus, Web of Science, Google scholar and BASE will be conducted from inception to present. Reference scanning and contact with content experts will be employed. Title/abstract screening and full text selection will be executed by two reviewers independently. Discrepancies will be resolved by discussion between reviewers or through third party intervention. Data will be extracted by a single reviewer and verified by a second. Clinical signs and symptoms data will be presented in terms of study design, setting and participant demographic data.

**Discussion:** Understanding biofilm impact on chronic wounds is inconsistent and based largely on *in vitro* research. This work will consolidate clinical signs, symptoms and biomarkers of biofilm in chronic wounds reported in the literature.

## Introduction

Wound healing occurs via a complex sequence of events which, under normal circumstances, proceed in an orderly fashion through haemostatic, inflammatory, proliferative and remodelling/maturation phases to restore cutaneous integrity and barrier function. However, in the presence of complicating factors such as diabetes or chronic venous insufficiency, the healing process can break down and the wound becomes chronic, failing to heal in a timely manner. ^1, 2^ There is a lack of consensus regarding the definition of chronic wounds and they have for example, been described as ‘wounds that have not proceeded through an orderly and timely reparation to produce anatomic and functional integrity after 3 months’, ‘wounds that lack a 20–40% reduction in size after 2–4 weeks of optimal treatment or when there is not complete healing after 6 weeks’, or simply as ‘wounds that fail to proceed through the normal phases of wound healing in an orderly and timely manner’ ^2, 3^. Typically, these wounds include but are not limited to venous, diabetic and pressure aetiologies ^4^. Chronic wounds of mixed aetiologies were estimated to have a pooled prevalence of 2.21 per 1000 population in a 2019 meta-analysis of three studies, while a second meta-analysis from the same year including nine studies estimated the pooled prevalence of chronic leg ulcers to be 1.5 per 1000 population ^5^. Chronic wounds are burdensome to the individual in terms of finances and quality of life, and to healthcare systems. Unhealed diabetic foot ulcers (DFU), venous leg ulcers (VLU) and pressure ulcers (PU) cost the United Kingdom National Health Service (NHS) approximately £4 billion in 2017–2018, or an average of £6305 per patient. ^6, 7^

Infection commonly affects chronic wounds and is associated with poor clinical outcomes ^8^. The risk of hospitalisation for patients with a DFU is increased by a factor of 50 while the risk of lower-extremity amputation is 150 times higher if their wounds become infected. Development of infection in chronic wounds is a complex process. Multiple factors including virulence of colonising organisms, synergy between multiple microbial species, bioburden and host resistance interact with each other and determine whether a wound will progress from a non-threatening, contaminated/colonized state through to critically colonized or infected states. ^9^

Bacteria can manifest in wounds as a free-floating planktonic phenotype or as a sessile biofilm phenotype ^10^. Biofilms occur when microbial cells organise themselves into aggregates or communities encased in a self-produced polymeric matrix which typically attach to surfaces. Biofilms exhibit high levels of tolerance to antimicrobial agents and host defences ^11^. When they form in critically colonized chronic wounds, healing stalls and the wound remains stuck in the inflammatory phase ^12– 16^.

In 2017, a panel of specialists, chosen for their expertise in chronic wounds and biofilms, for their scholarly activity and publication record, issued a consensus document ^17^. The document aimed to clarify the role of biofilms in clinical practice, help clinicians to recognize biofilms in chronic non healing wounds and optimise patient management. A modified Delphi process was used to achieve consensus on a series of statements formulated to address issues in ten areas relevant to management of non-healing chronic wounds. Five-point likert scales of agreement (1 = disagree strongly – 5 = agree strongly) and ranking (1 = not important – 5 = most important) were used to score the statements. There was strong agreement (mean 4.0, standard deviation [SD] 0.82) that specific clinical signs and symptoms should be used to confirm presence of biofilm in the absence of diagnostic bedside tests. Clinical features, such as a recurring gelatinous material on the wound edge, have been proposed as surrogate markers of wound biofilm but there was weak agreement (mean 3.6, SD 1.5) that the clinical signs and symptoms that could indicate the presence of biofilm. Little is known about presentation and diagnosis of wound biofilms and knowledge of their characteristics is limited ^18^. Generally, biofilms are difficult to diagnose and currently no guidelines exist to help clinicians and microbiologists in diagnosis and treatment ^19^.

In addition, little quantitative work has been done with respect to clinical signs and symptoms of biofilm in chronic wounds, especially in human patients, and existing published research is mainly observational rather than incorporating more rigorous study designs ^12, 17, 18, 20, 21^.

For these reasons we suspect that a rigorous systematic review with a focused research question and strict criteria with respect to eligible study design may be too exclusive and fail to answer the research question.

A scoping review methodology will therefore be employed to identify any associated clinical signs and symptoms thought to determine the presence of biofilm in chronic wounds.

The research question for the study is: what clinical signs, symptoms and biomarkers are proposed within the literature to determine the presence of biofilm in chronic wounds?

## Methods

The Preferred Reporting Items for Systematic Review and Meta-Analyses extension for Scoping Reviews (PRISMA-ScR) statement, the Joanna Briggs Institute Manual for Evidence Synthesis will guide this work ^22, 23^.

### Inclusion criteria

Eligible participants will be adults (18 years +). Eligible wound aetiologies will be venous leg ulcers (VLU), diabetic foot ulcers (DFU), pressure ulcers (PU) and/or mixed arterial/venous leg ulcers (MAVLU), treated in any setting.

Databases will be searched from inception to present without limits on language. Study designs including but not limited to systematic reviews, randomized controlled trials (RCTs), controlled clinical trials, cohort studies, case series, case reports, letters to the editor with relevant data and editorials will be included. These articles must report any clinical any clinical signs, symptoms and/or biomarkers, validated or otherwise, thought to be associated with the presence of biofilm in chronic wounds.

### Exclusion criteria

Patients with wounds resulting from burns, malignant fungating wounds, wounds secondary to conditions such as rheumatoid arthritis or pyoderma gangrenosum are ineligible for this study. Pay-per-view articles will not be included.

### Search strategy

***Database searching.*** A search strategy will be developed in Medline ( Box 1) and adapted for use in Embase, CINAHL, Cochrane Central, Scopus, Web of Science, Google scholar and BASE.

This strategy will utilise controlled vocabulary and keywords associated with the concepts of biofilm and chronic wounds that are currently known to the authors and taken from eligible articles located through a preliminary search of PubMed and CINAHL. Boolean operators AND, OR and proximity operators will combine search terms in a manner that optimises efficiency of the strategy, ensuring that the maximum number of potentially eligible articles are captured, and that as much irrelevant material as possible is eliminated prior to screening.

**Box 1. Search strategy for the Medline database**

      1.      skin ulcer/ or leg ulcer/ or foot ulcer/ or diabetic foot/ or varicose ulcer/ or pressure ulcer/ or wound infection/ or diabetic neuropathies/ or peripheral nervous system diseases/ or wound healing/ or debridement/ or “wounds and injuries”/ or peripheral arterial disease/ or re-epithelialisation/

      2.      Diabetes Mellitus, Type 2/co [Complications]

      3.      ("peripheral arterial" adj2 disease*).tw.

      4.      ((chronic or skin or varicose or venous or leg or vascular or foot or diabetic or neuropathic or arterial or isch?emic or neuro-isch?emic or neuroisch?emic or pressure or decubitus or mixed or "mixed ?etiolog*" ) adj3 (ulcer* or wound*)).tw.

      5.      ((Preven* or care or heal* or nonheal* or non-heal* or "non heal*" or re-epitheliali* or reepitheliali* or surface* or "lower extremit*" or "lower-extremit*" or debrid* or manag* or bed or "hard to heal" or hard-to-heal or infect*) adj3 (wound* or ulcer*)).tw.

      6.      (bedsore* or "bed sore*" or bed-sore*).tw.

      7.      ("diabetic foot" adj3 (syndrome or prevention)).tw.

      8.      1 or 2 or 3 or 4 or 5 or 6 or 7

      9.      exp Biofilms/

      10.      biofilm*.tw.

      11.      9 or 10

      12.      8 and 11

      13.      Limit 12 to humans

***Reference scanning.*** Reference lists of included articles will be scanned to locate subsequent, potentially relevant articles.

***Content experts and organisations.*** Content experts and relevant organisations will be consulted to obtain information about unpublished or ongoing studies and where applicable, to request access to known but unavailable sources of evidence.

Search results will be exported to EndNote X9 ^TM^ for storage and to RAYYAN ^24^ for screening against eligibility criteria.

### Evidence screening and selection

***Level 1 screening (title and abstract screening).*** Pairs of researchers will independently screen titles and abstracts for inclusion according to the pre-determined eligibility criteria. A single failed eligibility criterion will be considered sufficient to exclude a study from this review. Discrepancies will be resolved by discussion between researchers in a pair. In cases where disagreements cannot be resolved, a final decision on the discrepancy will be made by a third party.

The screening process will be pilot tested on a random sample of 50 titles and abstracts.

***Level 2 screening (full text screening).*** Pairs of researchers will independently screen all located full text articles for inclusion into this review according to eligibility criteria. A single failed eligibility criterion will be considered sufficient to exclude a study from this review **.** Discrepancies will be resolved by discussion between researchers in a pair. In cases where situations disagreements cannot be resolved, a final decision on the discrepancy will be made by a third party.

This level of the screening process will be pilot tested on a random sample of 10 articles if available.

A chart of the screening and selection process detailing the flow of studies from the search to data extraction and including duplicate removal will be presented with the findings.

An appendix of excluded full -text articles will also be included along with reasons for exclusion. 

### Data extraction

A data extraction form will be developed *a priori* in Microsoft Excel (2016). The form will capture the following data:

- The form will capture the following data:Study data including authorship, year of publication, article type/study design, country of origin, setting and study objective.
- Participant data including sample size, age, gender.
- Study concept data i.e. reported clinical signs, symptoms and biomarkers of biofilm in chronic wounds.

Data will be extracted by a single researcher and verified by a second.

### Critical appraisal/risk of bias assessment

The aim of this review is to collate a comprehensive list of signs, symptoms and biomarkers used to indicate presence of biofilm in chronic wounds regardless of their level of refinement. Relevant data may be found in articles that span the evidence hierarchy and range from systematic reviews to opinion/editorial articles ^21^. For these reasons, risk of bias assessment of included articles will not be necessary.

### Data analysis, summary and presentation

Extracted data will be tabulated. Concept data will be presented in terms of country of origin, setting, study design, and in terms of participant data i.e. sample size, wound aetiology, age and gender. Data will be analysed with SPSS statistical package version 26. Demographic data will be presented descriptively in terms of mean and standard deviation or median and range.

## Discussion

Little work regarding biofilms’ impact on chronic wounds involving human subjects has been done and much of our clinical understanding is based on *in vitro* work. Clinician’s knowledge of research data and of the importance of biofilms in the management of non-healing chronic wounds is inconsistent ^17^. This review will for the first time, consolidate those signs, symptoms and biomarkers of biofilm in chronic wounds reported in the literature into one document which may serve to open an avenue for future clinical research in this area. 

## Dissemination

The findings of this scoping review will be published in a peer-reviewed medical journal.

The research team will also regularly update and disseminate project findings to key stakeholders, research colleagues, patient representatives and knowledge users.

## Study status

The review has not yet initiated.

## Data availability

No data are associated with this article.
