# Supplementary material for: A scoping review protocol to identify clinical signs, symptoms and biomarkers indicative of biofilm presence in chronic wounds
Source: HRB Open Res. 2021 Nov 23;4:71. Originally published 2021 Jul 8. [Version 2] doi: 10.12688/hrbopenres.13300.2 (PMC8847728; doi:10.12688/hrbopenres.13300.2)
Supplement: Peer review history

# Peer review: Reviewer response for version 2

**DOI:** 10.21956/hrbopenres.14586.r31438

**Published:** 2022-02-18

**Authors:** Luc Teot

**Affiliations:** 1 Department of Plastic and Reconstructive Surgery and Wound Healing, Montpellier University Hospital, Montpellier, France

The eligibility criteria no. 1 may avoid the inclusion of a group of articles where biofilm is not mentioned in the abstract, even if in the full text there is a part concerning biofilms. On the contrary "optimistic" colleagues pretending that more than 90% of the wounds are contaminated by a biofilm may be considered as an extrapolation on which it will be difficult to define a clear opinion. 'Biofilm' is a very fashionable word.

 The inclusion criteria defines the usual chronic wounds but exclude most of the well known biofilm colonised wounds like the post trauma, post infection, malignant fungating wounds or post burn wounds.

 Another potential criticism concerns the rejection of a rigorous systematic review, replacing it by a scoping review methodology which is not well defined. Is it a score like for local infection signs or is it a list of signs, symptoms and biomarkers? All together or dissociated? Which ponderation will be applied to each of them?

 Is this research sponsored by a company and which one?

Is the study design appropriate for the research question?

Partly

Is the rationale for, and objectives of, the study clearly described?

Yes

Are sufficient details of the methods provided to allow replication by others?

Partly

Are the datasets clearly presented in a useable and accessible format?

Partly

Reviewer Expertise:

Wound healing

I confirm that I have read this submission and believe that I have an appropriate level of expertise to confirm that it is of an acceptable scientific standard, however I have significant reservations, as outlined above.

# Peer review: Reviewer response for version 2

**DOI:** 10.21956/hrbopenres.14586.r31436

**Published:** 2022-02-15

**Authors:** Gregory Schultz

**Affiliations:** 1 Department of Obstetrics and Gynecology, College of Medicine, University of Florida, Gainesville, FL, USA

The study proposal addresses an important issue for the field of chronic wound care, specifically, how can clinicians reliably assess if a chronic wound contains bacterial biofilm communities as part of the bacterial bioburden in the wound bed. The proposed methods and data analysis techniques are sufficiently described and adequate to address the key questions and objectives. The results of the research will be published in a peer-reviewed medical journal and will make a substantial contribution to the field of clinical care for chronic skin wounds. Funding is recommended with high level of enthusiasm.

Is the study design appropriate for the research question?

Yes

Is the rationale for, and objectives of, the study clearly described?

Yes

Are sufficient details of the methods provided to allow replication by others?

Yes

Are the datasets clearly presented in a useable and accessible format?

Yes

Reviewer Expertise:

bacterial biofilms in chronic wounds and point-of-care detectors for biofilm in wound beds

I confirm that I have read this submission and believe that I have an appropriate level of expertise to confirm that it is of an acceptable scientific standard.

# Peer review: Reviewer response for version 1

**DOI:** 10.21956/hrbopenres.14477.r29794

**Published:** 2021-07-14

**Authors:** Karen Ousey

**Affiliations:** 1 Institute of Skin Integrity and Infection Prevention, School of Human and Health Sciences, University of Huddersfield, Huddersfield, UK

A clear well, written, and structured scoping review. The area of biofilms is relevant to clinical practice and will be of interest to the multi-disciplinary team. I am sure the outcome of the review will identify areas for future research. The rationale and objectives are described coherently and supported by an appropriate study design. The methods suggested will allow for replication. I am surprised the authors have decided to exclude pay to view papers as I am worried this will preclude a lot of papers that will be relevant to the review.

Is the study design appropriate for the research question?

Yes

Is the rationale for, and objectives of, the study clearly described?

Yes

Are sufficient details of the methods provided to allow replication by others?

Yes

Are the datasets clearly presented in a useable and accessible format?

Yes

Reviewer Expertise:

Skin integrity, wound infection

I confirm that I have read this submission and believe that I have an appropriate level of expertise to confirm that it is of an acceptable scientific standard.

# Response

**Published:** 2021-08-11

**Authors:** John Ivory

**Affiliations:** National University of Ireland, Galway, Ireland

Dear Professor Ousey,

 Many thanks for taking the time to review this protocol and for the valuable feedback.

 We will change our eligibility criteria to admit pay-per-view articles.
